# Comparison of maternal outcomes in caring by Doula, trained lay companion and routine midwifery care

**DOI:** 10.1186/s12884-023-05987-7

**Published:** 2023-10-31

**Authors:** Shirin Shahbazi Sighaldeh, Afsaneh Azadpour, Katayoun Vakilian, Abbas Rahimi Foroushani, Seyedeh Fatemeh Vasegh Rahimparvar, Sedigheh Hantoushzadeh

**Affiliations:** 1 Department of Midwifery and Reproductive Health, School of Nursing and Midwifery, Tehran University of Medical Sciences, Tehran, Iran; 2 Nursing and Midwifery Care Research Center, Tehran University of Medical Sciences, Tehran, Iran; 3 Breastfeeding Research Center-Family Health Research Institute, Tehran University of Medical Sciences, Tehran, Iran; 4 MSc in Maternal and Child Health, School of Nursing and Midwifery, Tehran University of Medical Sciences, Tehran, Iran; 5 Department of Midwifery, School of Medicine, Arak University of Medical Sciences, Arak, Iran; 6 Department of Epidemiology and Biostatistics, School of Public Health, Tehran University of Medical Sciences, Tehran, Iran; 7 Department of Midwifery and Reproductive Health, School of Nursing and Midwifery, Tehran University of Medical Sciences, Tehran, Iran; 8 Department of Obstetrics and Gynecology, Imam Khomeini Hospital, Tehran University of Medical Sciences, Tehran, Iran

**Keywords:** Midwifery care model, Doula, Lay companion, Routine midwifery care, Iranian women, Quasi-experimental study

## Abstract

**Introduction:**

The aim of this study was to compare maternal and neonatal outcomes in the care provided by Doula, trained lay companion, and routine midwifery care in the labor and obstetric units. In this study, only results related to maternal outcomes were presented.

**Method:**

This is a quasi-experimental study, which was conducted on 150 women with low-risk pregnancies who had been selected for vaginal birth at private clinics and public hospitals of Arak, Iran. Participants were divided into three groups, two intervention groups, doula and trained lay companion, and one control group, midwife’s routine care. The intervention groups, in addition to receiving routine care from the labor and maternity units, also received support and training by doula or a trained lay companion, but 50 the control group received only routine midwifery care. In the control group and the trained companion, the samples were taken from 10 clinics of different parts of the city by random sampling method using the SIB center system. Then, among selected numbers, we randomly selected samples for each group. But in Doula group, because of limited number of samples, convenience sampling was used and all women enrolled in doula care were included in the study until the number reached 50. In each group, outcomes such as the duration of active phase and second stage of labor, as well as the severity of pain, anxiety and maternal satisfaction with birth were measured and compared with other groups. Data were collected by a researcher-made checklist, the Spielberger’s State-Trait Anxiety Inventory (STAI), the Pain Visual Assessment Scale (VAS), and the Hollins Martin’s Birth Satisfaction Scale-Revised (BSS-R). Data were analyzed by SPSS-22 statistical software using Kruskal Wallis, Chi-Square, ANOVA and Fisher’s exact tests.

**Findings:**

Based on the results, the mean duration of active phase between three groups was 234.68 ± 118.74, 256.66 ± 108.75 and 279 ± 94.37 min, respectively (p = 0.022). Also, the mean duration of second stage in three groups was 10 ± 5.61, 10.35 ± 5.1 and 22.30 ± 75.57 min, respectively (p < 0.001). The difference between mean pain scores in the first, second, third, fourth and fifth hours was not statistically significant. The average difference in anxiety score in the two stages of labor was higher in the lay companion group, and this difference was statistically significant (p < 0.001); however, the level of satisfaction in doula group was higher compared to the lay companion and control groups (p < 0.00 1).

**Conclusion:**

According to present study, doula care has a greater effect on reducing the duration of labor than other care models. Based on the study, there was no statistically significant difference between the three groups in terms of variables such as the severity of labor pain. However, the level of anxiety of pregnant mothers in the group supported by lay companion was lower than the other two groups, which indicates the positive effect of mothers’ training on increasing maternal comfort and satisfaction. It is suggested that further research investigate the severity of labor pain in groups supported by different care models and also we recommend the use of lay companion’ support during childbearing of mothers who could not afford doula.

**Trail registration:**

This article has been registered in Iran’s Clinical Trial Center with the code: IRCT20230620058548N1. 2023/08/29.

## Introduction

Women’s experiences of pregnancy and childbirth and of the care during those events, is important in all cultural contexts [1]. WHO recommends that every woman be offered the option to experience labor and childbirth with a companion of her choice [2]. The involvement of patients and clients as active participants in health care instead of passive care recipients has increased over the past few decades [3, 4], and it is associated with increased satisfaction with healthcare services [5, 6].

One of the most important predictors of a woman’s satisfaction of her labor and childbirth is the quality and level of support she received from her partner and from medical and birth professionals (e.g., doctors, midwives, nursing staff, doulas) [7] and the evidence supporting the recommendation emphasizes continuous support during childbirth [5, 8]. Continuous support is defined as “continuous presence and support during labor and birth”. The person providing the support could have qualifications as a healthcare professional (nurse, midwife), training as a doula or childbirth educator, or be a family member, spouse/partner, friend or stranger with little or no special training in labor support” [5]. When there is continuity in communication, women could trust the midwives [9].

A doula refers to a “trained professional who provides continuous physical, emotional and informational support to a mother before, during and after childbirth to help her achieve the healthiest, most satisfying experience possible” [10]. Doula cannot play the role of a delivery agent and is not allowed to have any clinical interventions during or after the labor process [11]. Doulas also help mothers to feel more knowledgeable and capable, alleviating potential distress during the labor process [12].

Advantages of doula’s presence include shorter labor duration, higher probability of spontaneous vaginal birth, lower risk of interventions such as medication and forceps, less chance of cesarean section, low-grade Apgar, and hospitalization, higher chance of breastfeeding and greater satisfaction with childbirth experience [13–15]. According to the findings of Kozhimannil et al. in women with doula support, the risk of cesarean section is reduced compared to women without doula support, and this can improve quality and safety of labor and reduce its costs [16].

A ‘lay companion’ refers to a person supporting a woman throughout labor and childbirth who is not a healthcare provider, doula or other trained professional. In practice, a ‘lay companion’ typically refers to a woman’s partner, family member, or friend [17]. When a relative or friend who has a good emotional relationship with the pregnant woman accompanies her in the maternity ward without interfering with the birth process, she can play an effective role by providing help, support, comfort, reassurance, and personal experience of the birth. She can also take action and do physical activities such as moving the pregnant women during labor, helping to change her position, and touching, massaging, and cooling her, etc. This can make childbirth a more pleasant experience, reduce the number of cesarean sections and save the costs of health care in any country [18]. In a research on continuous support of women during childbirth, especially by someone other than hospital staff, Hodnett et al. reported the results such as reduced labor duration, lower need for oxytocin and pain relief, and increased satisfaction with labor experience [8].

Given the importance of presence of a professional and non-professional companion in labor and birth for emotional and psychological support of the mother and also, due to the lack of studies that have measured the impact of such presence on maternal outcomes, the present study was conducted to compare the presence of a female relative or friend who has a close and emotional relationship with the mother but does not have the necessary skills, and also a doula who does not necessarily have a close relationship with the mother but has the necessary skills. This study also compares the presence of these individuals and routine care given to mothers during labor in order to find out which method produces better maternal and neonatal outcomes.

## Materials and methods

### Type of study

This is a semi-experimental study conducted on two intervention groups and one control group. In this study, the maternal and neonatal outcomes of the care provided by a doula and a trained companion were compared with the usual midwifery care in the labor and birth unit. In this article, only the maternal outcomes are discussed and the neonatal outcomes will be discussed in another article.

### Participants

The study population included 150 pregnant women who attended the clinics of public and private hospitals of Arak, which is one of the largest cities in central Iran and is the capital of Markazi province. The area of ​​this city is about 98.7178 km^2^ and covers 42.4% of the total area of ​​the province. The population of this city is 591,737 according to the 2016 national census. This city has public maternity hospitals including Imam Khomeini, Taleghani and Ebne-Sina hospitals, which offer doula services if requested by the mother.

### Inclusion and exclusion criteria

Criteria for entering the study included: low-risk pregnant women aged 18 to 40 years, gestational age of 38 to 42 weeks based on accurate LMP or ultrasound, spontaneous onset of labor pains, and healthy, live, single fetus with display of head and normal cervical delivery conditions according to midwife or physician. The criteria for not entering the study included: any known physical disease related or unrelated to pregnancy, mental illness of mother, known fetal abnormalities, previous history of cesarean section or desire to have selective cesarean section, and history of giving birth to infant who weighs less than 2500 or more than 4000 g. The exclusion criteria included a reluctance to continue with the study.

### Sample size

According to previous studies Khavandizadeh Aghdam [19], and Samieizadeh Tusi [20] as well as Nelson’s neonatal book [21], the standard deviation of labor length is 42 minutes and the standard deviation of the anxiety score is 12.8 units. Based on these standard deviations, the volume of different samples was obtained, so that with 95% confidence and 80% test power, we supposed “25 minutes” as the difference in the average length of labor in two groups, and 10 points” as the difference in the average anxiety score. Then by the related formulas, two sample sizes have been calculated and the maximum sample size has been selected, that was 45 people for each group. In order to avoid non-response, it was decided to add 5 samples to each group, so a total of 150 mothers, babies and companions were finally studied.

### Method

The trained (lay) companion group contained 50 pregnant women with 35–37 gestational weeks confirmed by accurate LMP or ultrasound from 10 pre-natal clinics of Arak public hospitals including Arastoo, Imam Khomeini, Jahangiri, Zeinab Kobra, 30-metri Miqan, Gavkhaneh, Hejrat, Shohadaie Safari, Valiasr, and Karahrud. The centers were selected from different parts of the city with clients coming from all economic and cultural classes who receive free services. To select the samples, we entered the SIB system in the mentioned centers and extracted the list of pregnant mothers who had the inclusion criteria from January to March 2019. Then the mothers were asked to enter the study and those who agreed were randomly selected. We used simple random sampling for choosing mothers from these centers. We had a list of all mothers of this center (from 1 to 389), then a number for each mother was randomly generated by using Excel, and the 50 samples that we required (from number 1 to 50) were then taken. After this, the necessary information about the objectives and method of study were given to them. The mothers were asked to nominate a person of their choice to accompany them during the labor and birth. A trained person should have at least one experience of childbirth. After being selected by the mother and introduced to the research team, they were invited to participate in educational classes, during which they were taught how to care for the mothers during labor. They were also assured that they would be notified of the time of the classes. After sampling was completed in this group, two training sessions were designed and the time of these sessions was coordinated with the mothers and their companions. Since a number of samples were excluded from the study for reasons such as not attending the training sessions, same number of eligible people were randomly assigned to the study to prevent any problem in statistical analysis. The mothers of this group finally gave birth in Imam Khomeini and Taleghani hospitals, according to their choice (Sina Hospital is private and Imam Khomeini and Taleghani Hospitals are public).

In the sessions, topics such as stages of labor, symptoms of labor, measures needed to facilitate labor, and role of the companion before coming to the hospital including: helping a pregnant woman to go to the hospital if she sees any signs of labor, continuous presence beside the patient, gaining the trust, building a sincere relationship, listening to the mother’s concerns and worries, encouraging proper breathing techniques, using massage or applying cold or hot compresses (according to the mother’s wishes), teaching relaxation methods, giving simple and understandable information about the birth process, encouraging frequent emptying of the bladder, helping and encouraging mobility, changing the position during the labor and using the delivery ball if possible, keeping the mother informed of the progress and birth process, helping the mother to get comfortable, encouraging the mother to push when needed, boosting her mood, preparing the mother for childbirth, advising the mother to work with the birth agent and teaching her how to breathe properly, applying pressure when infant is coming out, giving information about the importance of skin-to-skin contact with the baby immediately after the birth and the importance of early breastfeeding, were taught.

At the last session, the mothers and their companions were asked to inform the researcher when they see signs of childbirth, so that coordination could be made with the delivery and obstetric unit of Imam Khomeini and Taleghani Hospitals to accept them. If a woman with gestational age of under 37 weeks entered the delivery phase, she was excluded from the study, although we did not encounter such a sample. Also, if a woman had dilatation of 3 cm or more (active phase), she was admitted to the hospital.

Doula’s group contained 50 pregnant women aged 35 to 37 years, who had been selected from the counseling centers of Madaryar and Mamaye Mehraban using convenience sampling. According to the instructions of the Ministry of Health, the cost of doula in these centers is 50,000 Tomans ($ 4.2) per hour. Most of the clients who receive doula services are nulliparous women, and women who deliver their second child are more likely to apply for these services due to their difficult previous labor. In these centers, a doula is considered for all mothers. Doula is one of the center’s staff who personally takes all responsibilities of a doula during labor, but the delivery is done by another staff member.

62 mothers who had been registered for doula were selected from three Taleghani, Sina and Imam Khomeini hospitals from July 2018 to June 2019. Like companion group, simple random sampling was used for choosing mothers for this group. After providing a list of all mothers of this center (from 1 to 62), we randomly generated a number for each mother by using Excel and then 50 samples were taken (number 1 to 50). Finally, our samples gave birth in Imam Khomeini and Sina hospitals, and this was their choice, and no restrictions were considered.

According to the coordination made with the doulas, they were supposed to accept their patients in the active phase in the hospital, inform the researcher, accept the admission of each patient in the center, and teach mothers according to their own routine during the labor.

Samples of the control group were from mothers who attended the Taleghani Hospital for prenatal care. Like doula and companion group, we used simple random sampling for choosing mothers for control group, from September 2018 to October 2018. Accordingly, a list of all mothers of this hospital who had our criteria (from 1 to 95) was provided, then we randomly generated a number for each mother by using Excel and 50 samples (number 1 to 50) were then taken from 95 mothers. Selected mothers were asked to inform the researcher to coordinate with the hospital as soon as the labor pains began. When the women were admitted to the hospital, they were cared for by midwifery staff as usual. In this case, 5–6 midwives care for 6 maternity beds in each shift, and midwives are not required to go to see a pregnant woman in her bed when they have nothing to do with her delivery. Since Taleghani Hospital is a teaching and referral hospital, samples of the control group were shared jointly by midwives and assistants. In both trained companion and doula groups, supportive care began at the time of admission to the hospital and continued uninterruptedly until delivery and up to one hour after delivery. We could not add an explanation to the summary due to word limitation.

### Data collection tools

Data collection tools included:

1. A researcher-made checklist containing questions related to demographic information, fertility history and childbirth outcomes including the duration of active phase of the first stage (from 3 to 10 cm dilatation) and the duration of second stage of delivery (from 10 cm dilatation to complete exit of placenta), which was completed by the researcher. To determine the scientific validity of the first part of the tool (information checklist), the content validity method was used. For this purpose, 10 faculty members of the School of Nursing & Midwifery of Tehran University of Medical Sciences were consulted and according to their comments and suggestions, the necessary corrections were made and the final version of the tool was prepared and used with the approval of the supervisor. Simultaneous assessment method was also used to examine the reliability of information registration form. For this purpose, the researcher recorded the results of clinical examinations of several patients and then a midwife was asked to do the same examinations. After this, the researcher compared both results and if the similarity between them was above 80%, the scientific reliability of information registration was confirmed.
2. The Spielberger. State-Trait Anxiety Inventory (STAI). In this tool the score of 20 to 31 indicates mild anxiety, 32 to 53 shows moderate anxiety, 64 and higher indicates relatively severe anxiety, and score of 76 and above indicates very severe anxiety. The validity and reliability of this questionnaire were confirmed in 1994 by Mahram who studied 600 people [22]. The questionnaire was completed in two dilatation times of 3–4 cm and 8–10 cm by the researcher through an interview with the mother, and then the difference between the mother’s anxiety score in both times was calculated.
3. Visual Analogue Scale (VAS) for pain which uses a 10 cm calibrated ruler. The score of ten indicates the most severe pain and score of zero indicates no pain. The amount of pain is determined by the patient using the ruler. In several studies, the scientific validity and reliability of this tool have been confirmed [23, 24]. In Iran the reliability of this scale with the correlation coefficient of r = 0.88 has been confirmed. The pain scale was presented to the mothers at the time of admission and every hour until the time of delivery, and they were asked to indicate the severity of their pain by selecting one of the numbers.
4. Maternal satisfaction rate was assessed 24 h after the delivery with the revised satisfaction birth questionnaire by Hollins Martin (BSS-R). The revised birth satisfaction scale (BSS-R) was designed in 2011 by Caroline Hollins Martin et al. This revised questionnaire includes 10 questions in 3 areas of mother’s satisfaction with the quality of care provided, her personality traits, and the anxiety experienced during labor and childbirth [25]. In Iran, Rahimi Kian et al. (2017) have validated this questionnaire [26]. To express their satisfaction, the samples responded by choosing the options: I disagree, I have no opinion, I agree and I agree very much. For these responses, a score of 0 to 4 was considered, respectively, based on the 5-point Likert’s scale.

### Data analysis

The data were analyzed after collection by SPSS statistical software [22] using Kruskal Wallis, Chi-Square, ANOVA and Fisher’s exact tests. ANOVA method was used to moderate the effect of factors whose distribution was not homogeneous in the three study groups.

### Ethical considerations

The proposal of this project was approved by the Ethics Council of Arak University of Medical Sciences with the code of ethics: IR.TUMS.FNM.REC.1397.097. It was also registered in the Iranian Clinical Trials Registration Center.

### Findings

Study participants in the three groups of doula, trained companion and control, were in the age range of 23 to 30 years (60%, 58% and 52%, respectively). The majority of mothers in the three groups had diploma (82%, 64% and 42%, respectively). Most of the mothers were nulliparous (78%, 64% and 52%, respectively) and their infant weight was mainly between 2,500 and 4,000 g (92%, 98% and 96%, respectively). The demographic and midwifery characteristics of all samples are presented in Table 1. The companion chosen by the mothers in the trained companion group was one of the family members, including the mother or sister of the pregnant woman.

**Table 1:** Demographic characteristics and obstetric history of participants

Variable		Model of care in intervention and control groups			p-value
		Maternity ward midwife n (%)	Trained companion (family member)	Doula	
**Age (year)**	18–22 23–30 31–38	13(26) 26(52) 11(22)	12(24) 29(58) 9(18)	5(10) 30(60) 15(30)	0.222
**Educational level**	Illiterate & elementary/secondary school Diploma Bachelor’s degree and higher	10(20) 19(38) 21(42) 0(0)	5(10) 13(26) 32(64) 0(0)	1(2) 7(14) 41(82) 1(2)	0.001*>*
**Place of residence**	City Village	24(48) 26(52)	45(90) 5(10)	44(88) 6(12)	0.001*>*
**Parity**	First Second Third	26(52) 15(30) 9(18)	32(64) 13(26)) 5(10)	39(78) 10(20) 1(2)	0.054
**Infant’s birth weight(gr)**	2500*>* 250–4000 4000&more	1(2) 48(96) 1(2)	0(0) 49(98) 1(2)	1(2) 46(92) 3(6)	0.687

The average duration of active phase of labor was 279 ± 94.37 min in the control group, 234.68 ± 118.74 min in the doula group, and 256.66 ± 108.75 min in the lay companion group. The results of Kruskal Wallis test showed no statistically significant difference between the three groups in this regard (p-value = 0.063). However, when the relationship between the groups and the duration of active phase of labor was evaluated by ANOVA test and also confounding factors were controlled, this relationship was significant (p-value = 0.022).

The mean duration of second stage of labor was 22.75 ± 30.57 min in the control group, 10 ± 5.61 min in doula group and 10.35 ± 5.1 in the lay companion group. The difference between these variables was statistically significant. Therefore, the duration of second stage of labor was completely affected by the type of group (p-value < 0.001), (Table 2).

**Table 2:** Comparison of duration of first and second stage of labor in three study groups

Variable	Maternity ward midwife	Trained companion	Doula	p-value
**Active phase duration(minute)**	279 ± 94.37	256.66 ± 108.75	234.68 ± 118.74	0.063
** s stage duration(minute)**	22.75 ± 30.57	10.35 ± 5.1	10 ± 5.61	0.001>

In order to measure the severity of pain, considering that most samples (101 people) gave birth during the first 5 h of intervention (the severity of pain was not calculated for those who had cesarean section), the test could not be carried out between the three groups after the fifth hour, so the pain was measured at admission and then 1, 2, 3, 4, and 5 h after the admission. Comparing the severity of labor pain in the three study groups showed that, the mean pain score at the time of admission was 60.21 in the control group, 71.14 in the doula group and 62.69 in the trained companion group based on the VAS scale and the difference between these scores was not statistically significant. The mean pain score in the first hour after the intervention was 67.80 in the control group, 67.51 in the doula group and 59.52 in the trained companion group, and the difference between these scores was not statistically significant. The difference between mean pain scores in the second, third, and fourth hours was also not statistically significant. It means that, the severity of pain in the fourth hour of labor was not affected by the type of group and the care model (Table 3). However, the mean pain score in the fifth hour after the intervention in the control group was 33.85, in the doula group it was 20.93 and in the trained companion group it was 19.81, and the difference between these scores was statistically significant. Also, when four-variate ANOVA test was used to evaluate the relationship between the groups and severity of pain at the fifth hour and also the confounding factors (place of residence, education, and number of pregnancies) were controlled, statistically significant difference was not obtained for this relationship (p-value = 0.242).

**Table 3:** Comparison of the pain severity in the three study groups

Variable	Maternity ward midwife	Trained companion	Doula	p-value
Severity of pain at admission	60.21	62.69	71.14	0.337
Severity of pain in the first hour of labor	67.80	59.52	67.51	0.505
Severity of pain in the second hour of labor	67.51	57.49	59.78	0.409
Severity of pain in the third hour of labor	62.40	48.96	47.60	0.07
Severity of pain in the fourth hour of labor	44.29	36.91	33.68	0.176
Severity of pain in the fifth hour of labor	33.85	19.81	20.93	0.003

Comparison of the difference between anxiety score of mothers in two stages of labor in the three groups showed that, the mean difference in anxiety score in two stages of labor in the control group was 33.53, in the doula group it was 74.51 and in the trained companion group it was 80.08 and also the difference in these scores was statistically significant (p < 0.001) (Table 4). Therefore, the level of anxiety during labor was completely influenced by the type of group and the care model.

**Table 4:** Comparison of the mean anxiety score in two stages of labor in the three study groups

Variable	Maternity ward midwife	Trained companion	Doula	p-value
Manifest anxiety of mothers	33.53	80.08	74.51	< 0.001

For satisfaction with delivery in the three study groups of control, doula and trained companion, Kruskal Wallis test was used, the results of which are shown in Table 5. The mean score was 51.94 in the control group, 89.11 in the doula group and 85.45 in the trained companion group. The difference in these scores was statistically significant (p < 0.001) (Table 5). Therefore, the condition of maternity satisfaction was completely affected by the type of group and the care model.

**Table 5:** Comparison of satisfaction with delivery in the three study groups

Variable	Maternity ward midwife	Trained companion	Doula	p-value
Satisfaction	51.94	85.45	89.11	< 0.001

## Discussion

In the present study, which was conducted at the public and private hospitals and health centers that offer maternity services in Arak, the effect of care by doula and trained companion on maternal outcomes was evaluated and compared with routine midwifery care. The maternal outcomes investigated in this study included the duration of active phase and second stage of labor, the severity of labor pain, the degree of anxiety during labor and delivery, and finally, the mother’s satisfaction with the delivery process.

In relation to the average duration of active phase of labor, the results of present study showed that, the lowest mean duration of active phase of labor was related to the doula group, the trained companion group and then, the control group, respectively.

A study by Hodnett et al. (2013) to investigate the effects of continuous support during labor and delivery showed that ongoing supportive care for a pregnant woman compared to normal care can reduce the duration of labor [8]. Continuous support during labor is said to improve maternal and neonatal outcomes, including spontaneous vaginal delivery, shorter delivery times, reduced cesarean delivery and instrumental delivery, and reduced use of analgesia [5].

Studies by Bolbol Haghighi (2016), Landgreen (2010), and Safarzadeh et al. (2012) also showed that the presence of trained companion had a positive effect on the duration of active phase of labor [27–29]. In the study of Berghella et al. (2008), the duration of active phase of labor was lower in the group supported by doula than in the control group. According to this study, the presence of a supportive person such as patient’s mother, sibling or partner also reduced the duration of labor [30]. These results are not in agreement with the results of the studies of Brugman (2007) and Hatem (2008), which had found that the continuous supportive care by midwives failed to make a significant change in the mean duration of labor [31, 32]. One possible reason for these contradictive results could be the differences in the method and environment of studies. According to available literature, being in the correct position during labor with vaginal birth in addition to reducing labor pain, increases the chance of spontaneous childbirth, and also anatomically and psychologically promotes compatibility with the labor. It also reduces maternal fatigue, improves blood circulation in the mother and fetus, creates stronger and more effective uterine contractions, and reduces the duration of first and second stages of labor. Frequent changes in the state of mind by distracting the mother from pain, in addition to reducing the feelings of fatigue, facilitate childbirth and improve labor without the need for pharmacological intervention [33]. Midwives were unable to be continuously present [28, 34]. Unlike doulas, midwives perform multifaceted roles [34]. A midwife provides comprehensive care to women but might be unable to provide individualized continuous support unlike lay support persons who can offer continuous support [35]. Women’s preferred type of support persons was influenced by personal relationships, culture or birth setting. Lay support persons play a vital role which midwives cannot always fulfil due to their multiple roles. Conclusively, support persons do not replace the midwife’s role but rather complement supportive care [35]. In the present study, it seems that trained companion and doula, by accompanying and encouraging the mother to move and change position continuously during labor, were able to reduce the duration of active phase of labor.

Also, in the present study, the mean duration of second stage of labor in the three groups was compared with each other. The results showed that, the shortest duration of second stage of labor was related to the group supported by doula and after that, the group supported by the trained companion and then the group that received routine midwifery care. The difference between these variables was statistically significant. Therefore, the duration of second stage of labor was completely affected by the type of care. The second-stage of labor is defined as beginning with complete dilation of the cervix (10 cm) and ending with expulsion of the fetus [36]. Maternal and neonatal complications which happened during this period may be life threatening. For this reason, there is a necessity to manage the second-stage of labor in order to orchestrate safe vaginal deliveries. A study by Paterno et al. (2012) showed that, the presence of doula in childbirth shortens the duration of different stages of labor [37]. In a study by Shahshahan et al. (2014), 100 pregnant women were assessed in four groups of routine intervention with the presence of companion (family members or same-sex friend), routine intervention without companion, presence of companion without routine intervention, and lack of routine intervention or absence of companion. The results showed that, the presence of a supportive person during childbirth in Iranian women reduced the duration of first and second stages of labor and improved the outcomes of labor [38].

According to previous studies, moving and constantly changing the mother’s position during labor can shorten the duration of second stage of labor through several mechanisms such as: creating stronger and more effective contractions of the uterus and reducing the duration of labor, increasing the center of gravity and helping the fetus to rotate, and completing and facilitating the fetal movements. On the other hand, upright positions increase the pelvic outflow by 30 to 38% and shorten the delivery process. They also increase the force of gravity to move the fetus downward, thus helping the fetus to descend [33]. Chang et al. suggested that, the use of delivery ball causes more effective contractions and reduces the duration of labor [39]. In the present study, it seemed that the reduction in duration of second stage of labor was due to the same interventions and the delivery companions whether midwife or non-midwife (by encouraging the mother to move and change position during labor and delivery, and especially using the delivery ball) were able to reduce the duration of the second stage of the labor. On the other hand, in Bolbol Haghighi’s (2016) study, the mean duration of second stage of labor was lower in the group supported by trained midwifery students than in the control group, but this difference was not significant [27]. Burggemann et al. (2007) and Khresheh et al. (2010) showed no difference in the duration of second stage of labor in the two groups [31, 40]. This difference in results can also be due to differences in the research environment and how the intervention variables were controlled.

In the present study, the severity of pain has been measured from the time of admission to the fifth hour of each labor. The results showed that, the severity of pain was the same in all three groups. In other words, the severity of pain was not affected by the type of group. Lally et al. (2008) argued that a positive attitude, believing in the purposefulness of labor pain, distracting the mind from the pain, and thinking about the good days could decrease the intensity of labor pain [41]. The study of Darvishi et al. (2017) with the aim of investigating the effect of presence of midwives and non-midwives delivery agent on the rate of labor pains in nulliparous women showed that, the mean scores of pain in all three groups did not have a significant difference in the beginning of active phase, end of active phase and the second stage of delivery [42]. Studies of Nobakht et al. (2012) and Brugman et al. (2007), just like present study, showed that doula support does not affect variables such as reducing the severity of labor pains [31, 43]. However, a study by Ravangard et al. (2017) showed that in women accompanied by doula, the severity of pain was significantly reduced compared to the unaccompanied women [44]. Mothers could better deal with labor pain in the presence of their husbands or family members, and of course, a professional health care provider [45].

Differences in the results of these studies are probably due to individual differences in pain perception or the quality of maternity care provided by the companion. It is also possible that the quality of support provided by the midwives of the delivery unit was such that, it was able to reduce the differences between the three groups. This difference can be influenced by psychological, cultural, social, and environmental variables associated with pain behaviors [46].

In the present study, a comparison of maternal anxiety in the three study groups showed that, the average difference in anxiety score in the two stages of labor was higher in the trained companion group then doula and routine midwifery care groups, and this difference was statistically significant. The studies, like the present study, show that presence of an attendant or relative reduces the mother’s anxiety and fear and increases her comfort during labor and birth [43, 44]. A study by Brugman et al. in Brazil also showed that the presence of a companion during childbirth increases the satisfaction of mothers by reducing anxiety and fear [51]. Salehi et al.’s (2016) study was conducted on three groups of without support, supported by doula and supported by trained spouse. Based on the results of this study, the presence of trained spouses during childbirth was able to reduce women’s anxiety even more than doula [47]. Also, the findings of other studies, which assessed the effect of the presence of doula on anxiety, were consistent with the results of this study and showed that women accompanied by doula had significantly lower level of anxiety than those who were not accompanied by doula [12, 44, 48]. Doula support before and after birth can have a positive impact on maternal emotional wellbeing, by reducing anxiety, unhappiness and stress, and increasing self-esteem and self-efficacy [12].

In response to fear and anxiety, the level of catecholamines or stress hormones in the blood increases, causing a decrease in the level of oxytocin which results in decreased uterine contractions and prolonged active labor phase, and also a reduction in endorphins that increases the pain. Endorphins or encephalin are natural morphine-like proteins secreted by the hypothalamus and posterior pituitary. The more positive the mother’s attitude toward pain of childbirth is, the lower her fear would be and also endorphins would be secreted more, increasing its level in the blood. Massage activates the parasympathetic system, resulting in the production and release of endorphins which cause relaxation. The use of topical heat and skin stimulation (hot compresses and hot showers) reduces the level of catecholamines and increases endorphins [33]. These interventions were used in this study. Since the trained companion chosen by mother was more effective than doula in reducing maternal anxiety during labor and delivery, the use of such interventions would also be more effective. On the other hand, emotional closeness to the child and the assurance that comes from the presence of a compassionate and familiar person who can help the mother in all stages of labor can justify this reduction in anxiety of mothers. Numerous studies have already shown that if mother’s supporter is not a nursing or midwifery staff, she can better communicate with her and provide her with adequate support, such as doula’s support.

In the present study, the presence of doula was able to increase women’s satisfaction with childbirth, so that the highest level of satisfaction with childbirth was related to the doula group, followed by the trained companion and control group, respectively.

When comparing the results of the present study with other studies focusing on women’s birth satisfaction, we found some similarities. Almost all studies, including the present study, reported that, women who benefited from the presence of a companion were more satisfied with their delivery. We note the importance of the person providing the support in terms of increased women’s satisfaction with the birth experience. Maternal satisfaction includes all dimensions of care including: physical environment, cleanliness, availability of resources, interpersonal behavior, privacy, promptness, cognitive care, perceived provider competency, and emotional supports [49]. Similar to the present study, in some studies, the presence of doula as a lay companion increases maternal satisfaction with the experience of childbirth [14, 28, 50, 51]. Providing continuous doula program to pregnant women produces highest level of satisfaction [43]. Khresheh et al. (2010) compared women who received support from their relatives during labor with a control group (women who were only accompanied by medical personnel). Although the length of labor was similar in the two groups, those supported by their family needed lower amounts of analgesics and had a more pleasant experience during labor [40]. Green et al. (2014) showed that, the presence of doula as a lay companion increases maternal satisfaction with the experience of childbirth [14]. Women believed that the presence of a companion, e.g., their husband, a family member, or a doula, during labor helped them better deal with the labor process, particularly when they felt lonely [52]. In Yuenyong’s study (2012), the presence of a close relative of the pregnant woman during labor was able to reduce the duration of labor, and these women were more satisfied with their delivery [53]. In Brugmann study (2007), the presence of a companion chosen by the mother was able to produce a positive experience of the birth process [31]. In studies conducted by Gruber (2013) & Bruggemann (2007) and Gonzalez et al. (2010), women who were supported by lay companion were also more satisfied with the delivery than control group [31, 54, 55]. Studies by Sandall (2016) and Lundgren (2010) found that women who received continuous midwifery care were less likely to have interventions and were more satisfied [28, 51].

The findings of these studies on maternity satisfaction are consistent with the present study and this similarity is probably due to the similarities in study method and support of lay companion, which include both emotional and physical support. According to studies, several factors are considered as a measure of satisfaction, including quality of care, physical environment, effective communication, respect and security and comfort [56]. Vakilian et al.(2018) reported that midwife’s attitudinal skills has a positive influence on the experience of labor [57]. In the present study, the presence of lay companion (midwife or non-midwife) was able to increase maternal satisfaction with childbirth. Since the level of satisfaction in doula group was higher than the lay companion group, it is likely that doula’s higher skill in childbirth and providing services with a higher level of quality could be more effective in satisfying the mothers. Field et al. (1997) concluded in their study that the effect of childbirth support on maternity satisfaction was unclear and there was no significant difference between the two groups [58]. The reason for the differences in these results is probably due to the quality of delivery care provided by delivery companions and differences in measuring instruments.

Further studies with a larger sample size are suggested.

## Conclusion

According to present study, the mean duration of active phase and the second stage of labor were significantly different between the three groups of doula, lay companion and control group, and the shortest duration of labor was related to the doula group. This suggests that doula care has a greater effect on reducing the duration of labor than other care models. Also, the highest rates of maternity satisfaction were in the doula group, lay companion, and the group receiving normal care, respectively. On the other hand, the level of anxiety of pregnant mothers in the group supported by lay companion was lower than the other two groups, which indicates the positive effect of mothers’ training on increasing maternal comfort. Therefore, it can be said that due to the positive effects that a trained companion can have on the maternal and birth outcomes, it is recommended that mothers who do not have the ability to pay for doula care should be using the support of trained companion. It is also recommended that, this person along with the mother should take part in the maternity classes from the third trimester of pregnancy. On the other hand, based on the results of present study, there was no statistically significant difference between the three groups in terms of variables such as the severity of labor pain. It is suggested that further research should investigate the reason for this finding, and why there is no difference in the severity of labor pain in groups supported by different care models.

### Weaknesses and strengths

This study was initially designed as a randomized trial, but when we got to the sampling stage, we found that due to the small number of centers and the low number of mothers who want to receive support from a midwife, it is not possible to randomly allocate the samples in different groups, therefore, the study was conducted semi-experimentally, so that only random sampling was performed; but the allocation of samples into groups was not done randomly, and this could be one of the limitations of this study. One of the strengths of this study is the comparison of the effect of care provided by doula and the trained companion, which proved that the trained companion can have a positive effect on the mother’s satisfaction with the process of labor and childbirth, promotion of vaginal birth, and enhance mothers’ perception of quality of childbirth. Given that the cost of doula cannot be paid by families with poor socioeconomic status, our study proved the effectiveness of support by lay companion, so we recommend the use of these individuals in childbirth.

### Implications

The findings of this study showed that during the stressful time of the labor the presence of doula and lay companion might reduce the duration of labor, decrease the maternal anxiety, increase maternal satisfaction, increase the chance of skin-to-skin contact and early initiation of breastfeeding. Besides, it is worth noting that applying the inexpensive and easily accessible intervention that we reported and its effect on improving some main maternal outcomes during labor and delivery, lay companion, is in line with the policy of WHO and the Ministry of Health of Iran that is “to promote vaginal delivery and use of non-pharmacological methods of reducing labor pain”. Moreover, there is a growing desire to seek help from midwives and peers among Iranian mothers and other mothers all around the world, and among policy makers, as a factor that can have a positive impact on the quality of services in labor and delivery. Thus, the results of such studies can lead to reassuring mothers and policy makers, as well as encouraging policy makers to facilitate the use of lay companion during labor and delivery. Also, covering doula services in the list of insurance services can reduce the cost of using doulas and allow mothers and babies to benefit from their presence in labor and delivery.

## Data Availability

All data generated or analyzed during this study are included in this published article.
